# The White Clover TrMYB33-TrSAMS1 Module Contributes to Drought Tolerance by Modulation of Spermidine Biosynthesis via an ABA-Dependent Pathway

**DOI:** 10.3390/ijms25136974

**Published:** 2024-06-26

**Authors:** Youzhi Zhang, Xiaofang Qin, Zhirui He, Yan Zhang, Zhou Li, Gang Nie, Junming Zhao, Guangyan Feng, Yan Peng

**Affiliations:** College of Grassland Science and Technology, Sichuan Agricultural University, Chengdu 611130, China; b20172903@stu.sicau.edu.cn (Y.Z.); 2021202093@stu.sicau.edu.cn (X.Q.); 2021202091@stu.sicau.edu.cn (Z.H.); b20142403@stu.sicau.edu.cn (Y.Z.); zhouli2006@sicau.edu.cn (Z.L.); nieg17@sicau.edu.cn (G.N.); feng0201@sicau.edu.cn (G.F.)

**Keywords:** white clover, drought resistance, MYB transcription factor, S-adenosylmethionine synthase, spermidine, ABA dependent

## Abstract

Spermidine is well known to accumulate in plants exposed to drought, but the regulatory network associated with its biosynthesis and accumulation and the underlying molecular mechanisms remain unclear. Here, we demonstrated that the *Trifolium repens* TrMYB33 relayed the ABA signal to modulate drought-induced spermidine production by directly regulating the expression of *TrSAMS1*, which encodes an S-adenosylmethionine synthase. This gene was identified by transcriptome and expression analysis in *T. repens*. *TrSAMS1* overexpression and its pTRV-VIGS-mediated silencing demonstrated that *TrSAMS1* is a positive regulator of spermidine synthesis and drought tolerance. TrMYB33 was identified as an interacting candidate through yeast one-hybrid library screening with the *TrSAMS1* promoter region as the bait. TrMYB33 was confirmed to bind directly to the predicted TAACCACTAACCA (the TAACCA MYB binding site is repeated twice in tandem) within the *TrSAMS1* promoter and to act as a transcriptional activator. Additionally, TrMYB33 contributed to drought tolerance by regulating *TrSAMS1* expression and modulating spermidine synthesis. Additionally, we found that spermidine accumulation under drought stress depended on ABA and that TrMYB33 coordinated ABA-mediated upregulation of *TrSAMS1* and spermidine accumulation. This study elucidated the role of a *T. repens* MYB33 homolog in modulating spermidine biosynthesis. The further exploitation and functional characterization of the TrMYB33–TrSAMS1 regulatory module can enhance our understanding of the molecular mechanisms responsible for spermidine accumulation during drought stress.

## 1. Introduction

Plants are often subjected to various abiotic stresses, with drought being one of the most stress-inducing environmental conditions; it severely limits plant growth and development [1], as well as crop productivity and quality. When experiencing water scarcity, plants react by undergoing various physiological and biochemical adaptations to enhance their resistance to drought. Consequently, elucidating the changes that allow plants to alleviate drought-related damage will provide valuable knowledge for understanding the complex reprograming governing plant adaptation to and tolerance of drought stress.

Plants have developed the capacity to endure drought stress as a result of their adaptation to water scarcity. Transcriptional functional rearrangement of drought-responsive regulatory genes has been recognized as a key mechanism in this process [2,3,4]. Progress has been made in the identification and classification of molecules that are involved in the regulation of drought stress responses, especially regarding transcription factors (TFs) and their target genes. Research and findings by Chen et al. [5], Thirumalaikumar et al. [6], Ma et al. [7], Li et al. [8], and Ju et al. [9] have significantly contributed to this progress. MYBs, among other TFs, have been documented to play a significant role in regulating drought stress tolerance. MYB TFs are classified based on the number of repeats in their structure, which ranges from one to four. The TFs are also characterized by their DNA-binding domains. The most prevalent MYB TFs in plants belong to the R2R3-MYB subfamily and are crucial in drought stress responses [10]. Despite the limited number of 3R-MYBs, *Os*MYB3R-2 [11] and *Ta*MYB3R1 [12], members of this class, are involved in plant responses to drought stress. Based on transcriptomic studies in Arabidopsis thaliana, 51% of *At*MYB genes are upregulated [13] under drought stress. Additionally, MYBs have been functionally characterized and were shown to be involved in drought stress responses [14]. *AtMYB77* [15] and *AtMYB60* [16] are involved in lateral root growth. *Arabidopsis* plants overexpressing *AtMYB60* exhibited increased root mass when grown on MS plates containing mannitol. Silencing *NbPHAN* (a member of the R2R3-type MYB subfamily) altered the leaf shape, caused ectopic growth, and reduced the expression of drought-related genes. All these genes were highly expressed under water-deficit conditions. Although some studies have suggested MYBs’ involvement in the drought response, the regulatory network and the target gene (s) of MYBs in response to drought stress remain unclear.

Furthermore, MYBs also function as master regulators to coordinate ABA-mediated responses. For example, *At*MYB96 is a crucial factor that coordinates ABA signaling to govern lateral-root development in water-limited environments, and its expression is considerably induced by ABA [17]. On the other hand, ABA treatment down-regulated *VvMYB60* expression [18]. *AtMYB20*-overexpressing *Arabidopsis* displayed no sensitivity to ABA and heightened susceptibility to desiccation, while increased ABA sensitivity and resistance to desiccation were observed in *AtMYB20* knock-out plants [19]. AtMYB61 also appears to regulate stomatal aperture in an ABA-independent manner [20]. However, the networks coordinating ABA and MYBs in drought responses require further investigation.

Many genes are expressed under water-deficit conditions in plants [21], and some participate in the biosynthesis of protective metabolites known as polyamines (PAs) [22]. PAs are beneficial to plants, as they promote protein homeostasis and detoxify reactive oxygen species (ROS) [23]. Indeed, the levels of PAs have been shown to significantly increase in plants exposed to drought. These changes primarily affect PA metabolism and other signaling pathways [24,25]. Putrescine (Put), spermidine (Spd), and spermine (Spm) are the most abundant PAs. In plants, ornithine or arginine undergoes decarboxylation, resulting in Put formation. The amino-propyl group, donated by S-adenosylmethionine (SAM), which is produced by S-adenosylmethionine synthase (SAMS), is then added to Put to generate Spd and Spm sequentially [26,27]. The plant SAMS genes that have been characterized to date have been shown to be strongly upregulated by drought stresses [28,29,30] and function in PAs synthesis [31,32,33]. However, our knowledge of the SAMS genes is still incomplete, as only a small number of SAMS genes have been functionally characterized for their involvement in drought stress tolerance. Additionally, only a few of the upstream regulators of SAMS genes have been identified. The signal-transduction pathway and regulatory network connecting the drought stimulus to polyamine biosynthesis also remain unclear. It is important to note that there is a missing link between MYB expression and the accumulation of PAs, despite their individual correlations with drought responses.

White clover (*Trifolium repens*), a perennial legume grass commonly utilized for grazing and lawn greening, predominantly has poor drought tolerance [34,35]. As a result, it is a promising candidate for examining drought-response mechanisms and identifying functional genes for the genetic improvement of legumes. In our previous work, we performed RNA sequencing (RNA-Seq) analysis to understand the transcriptome dynamics between drought-tolerant and drought-sensitive genotypes in *T*. *repens*. This analysis revealed several differentially expressed genes (DEGs) associated with numerous metabolic pathways (not yet published). In this study, we identified a *T. repens* SAMS gene (*TrSAMS1*) and assessed its functions in drought tolerance. In addition, we demonstrated that a *T. repens* MYB33 TF (*Tr*MYB33) acts as a transcriptional factor, binding to *TrSAMS1* to regulate spermidine production under drought stress. Furthermore, our findings suggest that *Tr*MYB33 plays a crucial role in coordinating the ABA signal to control drought-induced spermidine accumulation. Considering this evidence together, we propose a regulatory module encompassing ABA-*Tr*MYB33-*Tr*SAMS1-Spd that facilitates drought-induced *Tr*SAMS1 upregulation and spermidine production. These findings shed light on the molecular events linked to spermidine accumulation in plants under drought stress.

## 2. Results and Discussion

### 2.1. Identification and Analysis of TrSAMS1 and Three Genes Involved in Spd Synthesis

In our previous research, exogenous spermidine (Spd) could improve photosynthesis and water-use efficiency and lower oxidative damage [36]. Additionally, spermidine-treated white clover had a higher abundance under drought stress of differentially expressed proteins involved in amino acid and protein biosynthesis, carbon metabolism, and antioxidant and stress defense, as well as in the ABA signaling pathways [36]. To identify the specific genes that were responsible for the increase in spermidine during drought, we analyzed the RNA-Seq dataset. We discovered three DEGs annotated as S-adenosylmethionine synthetase (SAMS, Unigene18063, CL11256, CL6473), one DEG annotated as S-adenosylmethionine decarboxylase (SAMDC, Unigene9738), and two DEGs annotated as spermidine synthase (SPDS, Unigene7182, Unigene21202), all of which were upregulated under drought stress (Supporting Information: Figure S1). As previously reported, white clover *TrSAMDC1* (Accession No. MN400662) was shown to mitigate the negative effects of drought stress by indirectly enhancing Spd content [37]. In this study, we evaluated *SAMS* and its associated transcription factor. Detailed information on SAMS was documented in our previous results [38]. In this study, we used four *SAMS* of *A. thaliana* and the white clover *SAMS* to construct a phylogenetic tree. It was observed that the gene was most closely related to *AtSAMS1* (Supporting Information: Figure S2); therefore, it was named *TrSAM1* (*T. repens SAMS1*). Based on the white clover genome-sequence information (retrieved from NCBI, https://www.ncbi.nlm.nih.gov/genome/13404 (1 May 2024)), *TrSAMSl* was located in tandem on chromosome 2P and lacked an intron (Supporting Information: Figure S3). Multiple protein-sequence alignments revealed that TrSAMSl shares high sequence similarity with SAMS proteins of *Oryza sativa* subsp. *japonica*, *Arabidopsis thaliana*, *Medicago truncatula*, and *Glycine max* (Supporting Information: Figure S4).

### 2.2. TrSAMS1 Promotes Spd Synthesis and Positively Contributes to Drought Tolerance in Arabidopsis and White Clover

Our previous findings suggested that *Tr*SAMS1 is localized in the nucleus [38]. We quantitatively assessed the relative expression of *TrSAMS1* in five *A. thaliana* overexpression lines (Supporting Information: Figure S5a). We additionally detected the NPT gene (NPT-F and NPT-R listed in s Table S1, 210 bp) in the plasmid backbone (Supporting Information: Figure S5b). Then, two transgenic lines, OE1 and OE3, which had the highest relative expression levels of *TrSAMS1*, were selected to assess plant drought tolerance.

**Figure 1:**
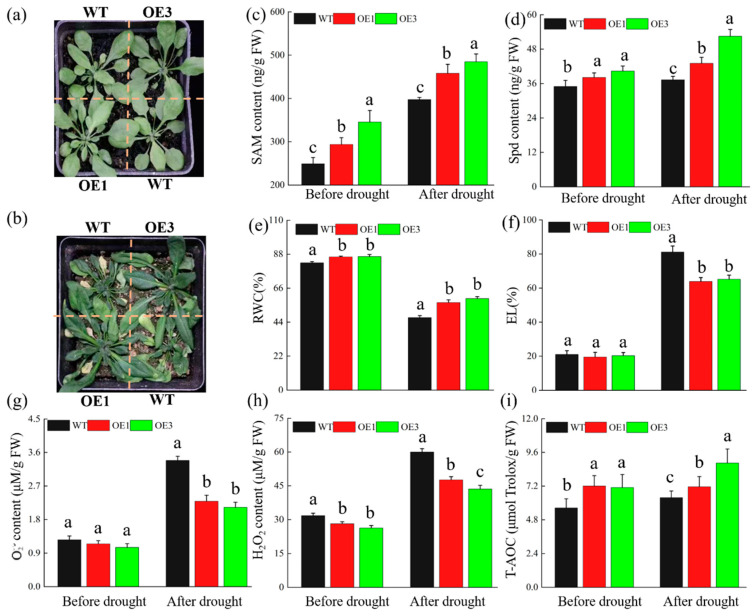
Overexpression of *TrSAMS1* confers enhanced drought tolerance in *A. thaliana*. (**a**,**b**) Phenotypes of transgenic and WT plants before (**a**) and after drought treatment (**b**). (**c**) SAM content of two transgenic lines (OE1 and OE3) and WT plants, measured before and after the drought treatment. (**d**) Spd content of two transgenic lines (OE1 and OE3) and WT plants, measured before and after the drought treatment. (**e**,**f**) RWC (%) (**e**) and EL (%) (**f**) of two transgenic lines and WT PLANTS, measured before and after the drought treatment. (**g**) $O_{2}^{-}$ content. (**h**) H_2_O_2_ content. (**i**) T-AOC. Error bars indicate standard deviation (n = 3). Different letters indicate significant difference (*p* < 0.05).

The phenotypes of the *A. thaliana* lines under normal conditions (Figure 1a) and under drought stress (Figure 1b) illustrated *TrSAMS1*’s effectiveness in promoting plant growth and enhancing drought resistance. The transgenic lines exhibited significantly higher levels of both SAM (Figure 1c) and Spd (Figure 1d) than did WT plants before and after exposure to drought, indicating that *TrSAMS1* overexpression increases their content. Additionally, there was a significant difference in relative water content (RWC, %) (Figure 1e) between WT plants and OEs before and after drought. However, electrolyte leakage (EL, %) (Figure 1f) and superoxide anion ($O_{2}^{-}$) (Figure 1g) content significantly decreased in OEs only after drought. Regarding hydrogen peroxide (H_2_O_2_) and total antioxidant capacity (T-AOC), there were significant differences between WT and overexpression plants under both normal growth conditions and drought stress (Figure 1h,i), indicating that *TrSAMS1* overexpression had a substantial effect on reducing H_2_O_2_ and increasing T-AOC levels in plants. These results suggested that *TrSAMS1* overexpression could significantly enhance drought tolerance in transgenic *Arabidopsis* plants.

**Figure 2:**
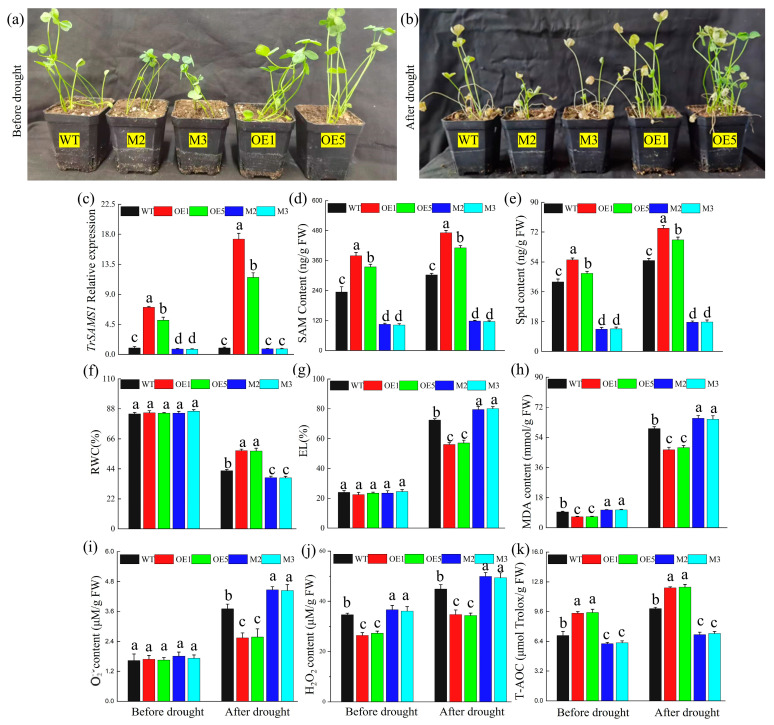
Overexpression of *TrSAMS1* confers enhanced drought tolerance in *T. repens*. (**a**) Phenotype of white clover before drought stress. (**b**) Phenotype of white clover after drought stress. (**c**) *TrSAMS1* relative expression. (**d**) SAM content. (**e**) Spd content. (**f**) RWC (%). (**g**) EL (%). (**h**) MDA content. (**i**) $O_{2}^{-}$ content. (**j**) H_2_O_2_ content. (**k**) T-AOC. Error bars indicate standard deviation (n = 3). Different letters indicate significant difference (*p* < 0.05).

Furthermore, we generated white clover plants overexpressing *TrSAMS1* and *TrSAMS1*-knockdown lines. Green fluorescence was emitted by calli of white clover overexpressing TrSAMS1 on exposure to 488 nm excitation light. (Supporting Information: Figure S6a). These calli were subsequently cultured for root generation (Supporting Information: Figure S6b). Two *TrSAMS1*-overexpressing white clover lines, OE1 and OE5, which had higher *TrSAMS1* expression levels (Supporting Information: Figure S6c) compared to the other lines, were experimentally assessed in this study. In the pTRV-VIGS *TrSAMS1*-knockdown white clover plants, the *TrPDS* fragment (Supporting Information: Figure S6d) and the *TrSAMS1* fragment (Supporting Information: Figure S6e) obtained through PCR were ligated to linearized pTRV2 vectors. Schematic diagrams of the vector constructs can be found in the supporting information (Supporting Information: Figure S7a). Seedlings inoculated with TrPDS-pTRV2 + pTRV1 exhibited photobleaching, as characterized by numerous white spots on the leaves (Supporting Information: Figure S7b); this phenomenon was not observed in the mock-treatment (Supporting Information: Figure S7c) or water control plants (Supporting Information: Figure S7d). For this study, two *TrSAMS1*-knockdown white clover lines, M2 and M3, which had the lowest *TrSAMS1* expression (Supporting Information: Figure S7e) were experimentally evaluated. *TrSAMS1*-overexpressing white clover plants were taller and exhibited increased drought resistance, with most leaves retaining their green color after 8 days of drought. The phenotypes of white clover plants before drought are shown in Figure 2a. The *TrSAMS1*-knockdown white clover plants exhibited weakness, with most leaves turning yellow after 8 days of drought (Figure 2b). Compared to the WT and the knockdown plants, the relative expression of the *SAMS* gene was significantly higher in OE1 and OE5 plants before and after drought stress (Figure 2c). The relative expression of the *SAMS* gene in M2 and M3 significantly differed from the WT plants before drought but not after drought stress (Figure 2c). 

Additionally, the SAM and Spd contents in OE1 and OE5 plants were significantly higher than those in WT and knockdown plants before and after drought. Moreover, SAM (Figure 2d) and Spd (Figure 2e) in WT plants were significantly higher than in the knockdown plants. Under normal growth conditions, there were no significant differences in RWC (Figure 2f) and EL (Figure 2g) among all plant lines. The RWCs of OE1 and OE5 were 34.79% and 34.21% higher, respectively than that of WT plants during drought, while the RWCs of the knockdown lines M2 and M3 were 11.85% and 12.29% lower than that of WT plants, respectively (Figure 2f). Furthermore, the EL values of OE1 and OE5 were 22.54% and 21.10% lower than that of WT plants during drought, while the EL values of M2 and M3 were 9.75% and 10.66% higher than that of WT plants, respectively (Figure 2g). Finally, significant differences were observed between WT plants and the OE and knockdown plant lines in terms of MDA content under normal growth and drought stress (Figure 2h). Under normal growth conditions, there was no significant difference in $O_{2}^{-}$ content among the plant lines. However, under drought conditions, WT plants exhibited substantial differences from the other plant lines (Figure 2i). During normal growth and drought stress, there was a significant difference in H_2_O_2_ content and T-AOC between WT plants and the OE and knockdown plant lines (Figure 2j,k). These findings suggest that increased expression of *TrSAMS1* is positively associated with plant resistance to drought. 

### 2.3. The TrMYB33 Transcription Factor Regulates TrSAMS1 Transcription

The *T. repens* cDNA library was probed to identify TFs that bind to the *TrSAMS1* promoter region using three baits through Y1Hgold. A total of 53 positive colonies were identified, and select inserts were shown to encode MYB proteins, including *Tr*MYB33. The sequences of all *A. thaliana* MYBs used for the generation of the phylogenetic tree were downloaded from the PlantTFDB website (http://planttfdb.gao-lab.org/family.php?sp=Ath&fam=MYB (accessed on 1 May 2024)). Phylogenetic analyses revealed that the identified *T. repens* MYB was most closely related to *A. thaliana* At5G06100s (Supporting Information: Figure S8a), all three of which corresponded to *At*MYB33. Consequently, the protein was annotated as *Tr*MYB33. Additionally, we compared the DNA sequence of *Tr*MYB33 with that of the white clover genome and discovered that its CDS matched sequences in the 3O and 3P chromosomes. (Supporting Information: Figure S8b,c).

**Figure 3:**
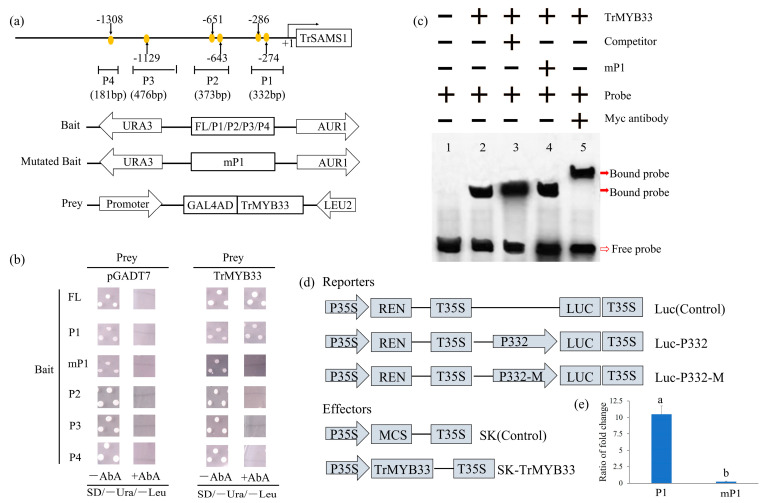
TrMYB33 binds to and activates the promoter of *TrSAMS1*. (**a**) Schematic diagrams of the *TrSAMS1* promoter, 1362 bp upstream of the translational start site (TSS, +1), and bait and prey constructs used for the yeast (Saccharomyces cerevisiae) one-hybrid assay. MYB binding sites are denoted using orange circles and numbered in the light of their distance from the TSS, which is shown at position + 1. FL, full length fragment. P1, P2, P3 and P4 are four fragments containing a MYB binding-site core sequence in each, while mP1 is mutated form of P1. (**b**) Growth of yeast cells transformed with different combinations of bait and prey on selective medium. (**c**) EMSA assays showing direct and specific binding of TrMYB33 to the MYB binding-site element in the *TrSAMS1* promoter. The bound-protein–DNA complex and the free probe are shown by the closed and open arrows, respectively. − and +, absence (−) or presence (+) of corresponding component shown above. (**d**) Schematic diagrams of effector and reporter constructs, driven by CaMV35S promoter, used for dual-LUC transient-expression assay. MCS, multiple cloning sites. P35S, the CaMV35S promoter. T35S, the CaMV35S terminator. LUC, firefly luciferase. REN, Renilla luciferase. (**e**) Dual-LUC expression assays in tobacco (*Nicotiana benthamiana*) cells using vectors in (**d**). Error bars represent ± standard deviation (n = 3). Different letters indicate that the values are significantly different from each other (*p* < 0.05).

Y1Hgold yeast cells co-transformed with the baits (constructed using FL, P1, mP1, P2, P3, and P4) (Figure 3a) and prey could grow on the SD/-Ura/-Leu medium (Figure 3b). Only the yeast carrying baits FL or P1 and TrMYB33 could grow on the SD/-Ura/-Leu/AbA (100 ng/mL) (Figure 3b). Subsequently, the MYB-binding sites in P1 were removed to construct mP1 and Y1Hgold yeast cells carrying mP1 and *Tr*MYB33 did not grow on the medium (Figure 3b). 

Then, we transferred the *Tr*MYB33-pGBKT7 recombinant vector to *E. coli* BL21 (DE3). The total proteins of BL21 were extracted, and an SDS-PAGE analysis was conducted to isolate the *Tr*MYB33 protein. A protein band ranging from 45 kD to 35 kD was identified, which was consistent with the expected properties of *Tr*MYB33 (41.24 kD, with a Myc tag of 1.2 kD.). Subsequently, we performed Super-shift EMSA to determine whether *Tr*MYB33 could directly bind to the MYB binding sites. Incubation of the *Tr*MYB33 protein (carrying a Myc tag) and the labeled probes resulted in a band shift (Figure 3c), indicating the direct and specific binding of *Tr*MYB33 to MYB binding sites within the P1 region of the *TrSAMS1* promoter. To further establish TrMYB33’s role in gene activation, we generated firefly LUC reporter constructs driven by the P1 fragment of the *TrSAMS1* promoter (Figure 3d), along with an effector. *Tr*MYB33 with the P1 fragment led to over 42-fold activation of LUC in comparison to TrMYB33 carrying the mP1 fragment (Figure 3e). These findings indicated that *Tr*MYB33 serves as a transcriptional activator of *TrSAMS1* via interactions with the MYB binding sites on the *TrSAMS1* promoter.

### 2.4. TrMYB33 Is Localized in the Nucleus and Has the Ability to Activate Transcription

**Figure 4:**
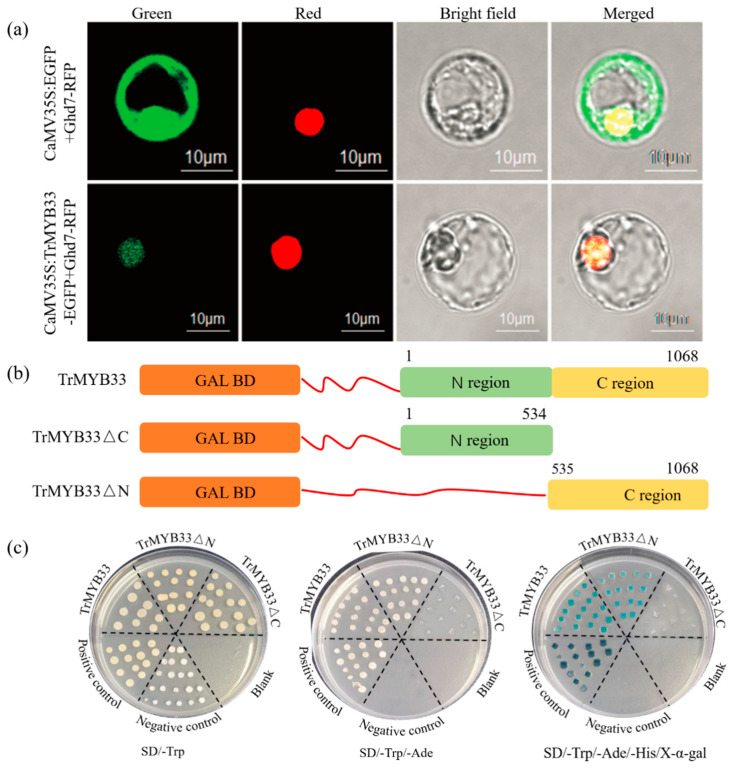
Subcellular-localization and transcriptional-activity assays of TrMYB33. (**a**) Subcellular localization of TrMYB33 based on visualization of EGFP (enhanced green fluorescent protein) signal. The fusion construct (Ca35S: TrMYB33-EGFP) or empty vector (35S:EGFP) was co-transformed with a nuclear marker gene, Ghd7-RFP, into *A. thaliana* leaves. Confocal microscopic images of epidermal cells were taken under green (for EGFP), red (LSD1-RFP) fluorescence, and bright-field illumination. The images on the right overlap those on the left. Bars, 10 μm. (**b**) Schematic diagrams of construct vectors used for transcriptional-activity assays. Full-length sequences and truncated fragments of TrMYB33 were introduced downstream of the GAL4BD (galactose-specific transcription enhancing factor 4 binding domain) in the pGBKT7 vector. TrMYB33ΔC and TrMYB33ΔN indicate removal of the C and N termini, respectively. The numbers above the CDS represent the position of the nucleotide residue. (**c**) Growth of AH109 yeast (*Saccharomyces cerevisiae*) cells transformed with the vectors, accompanied by a positive control (pGBKT7-53 + pGADT7-T) and negative control (pGBKT7), on SD/-Trp, SD/-Trp/-Ade, and SD/-Trp/-Ade/-His /X-α-gal.

To investigate the subcellular localization of *Tr*MYB33, we co-expressed CaMV35S: *Tr*MYB33-EGFP or CaMV35S: EGFP (control vector) in *A. thaliana* leaves. Microscopic observations revealed that the EGFP signal from the control vector spread throughout the cell. However, the EGFP signal from *Tr*MYB33-EGFP was observed in the nucleus and co-localized with the LSD1 nuclear marker, providing evidence that *Tr*MYB33 is a nuclear-localized protein (Figure 4a). To determine TrMYB33’s transcription-activation capacity, we conducted a transactivation reporter assay with three effectors (Figure 4b). As indicated in the domain analysis on InterPro, the MYB domain encompasses AA^18^ to AA^72^ and AA^75^ to AA^123^. AH109 yeast cells grew well on the SD/-Trp medium. However, only those transformed with constructs containing the full-length protein sequence and C region and the positive control exhibited normal growth on the selective media and displayed α-galactosidase activity (Figure 4c). These combined results indicate that TrMYB33 possesses a transcription-activating activity, with the activation domain located in the C region.

### 2.5. TrMYB33 Regulates Spd Accumulation and Contributes to Drought Tolerance in Arabidopsis

Since the *A. thaliana* MYB gene was found to be correlated with drought tolerance and *Tr*MYB33 was identified as an upstream regulator of *TrSAMS1*, it was hypothesized that *Tr*MYB33 could play a role in drought tolerance by regulating Spd biosynthesis and accumulation. *TrMYB33*-overexpressing *A. thaliana* plants (OEs) were generated and confirmed molecularly through the amplification of the NPT fragment (210 bp) using genomic PCR (Figure S9a). Two separate lines that displayed significantly higher TrMYB33 transcript levels, OE2 and OE8, were selected (Figure S9b). 

**Figure 5:**
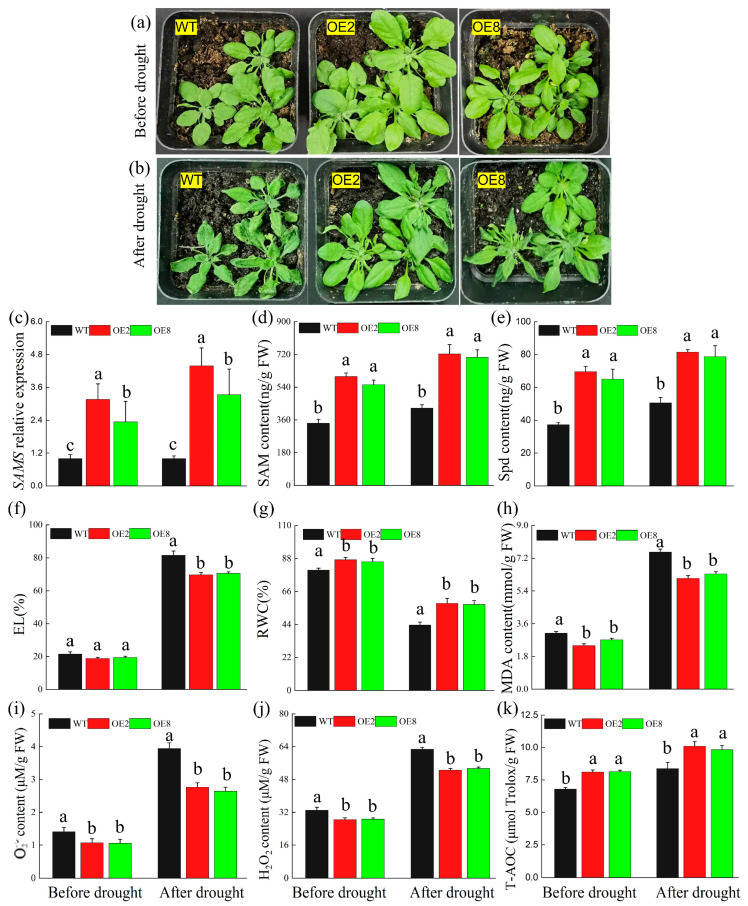
*TrMYB33* overexpression increases SAM and Spd production and confers enhanced drought tolerance in *A. thaliana*. (**a**,**b**) Phenotype of WT plants, OE2, and OE8 before (**a**) and after stress ((**b**), rewatering). (**c**) *SAMS* relative expression. (**d**) SAM content. (**e**) Spd content. (**f**) EL (%) of the WT and transgenic lines. (**g**) RWC (%). (**h**) MDA content. (**i**) $O_{2}^{-}$ content. (**j**) H_2_O_2_ content. (**k**) T-AOC. Error bars indicate standard deviation (n = 3). Different letters indicate significant difference (*p* < 0.05).

Notably, prior to the drought stress treatment, the OE lines displayed slightly increased growth compared to wild-type plants (Figure 5a). After drought treatment, the plants overexpressing *TrMYB33* displayed a more drought-tolerant phenotype compared to the wild type (Figure 5b). We examined whether *TrMYB33* overexpression modulated the biosynthesis and accumulation of SAM and Spd in *A. thaliana* by analyzing the relative expression of the *SAMS* gene and the SAM and Spd contents. The transgenic lines exhibited higher *SAMS* gene expression, SAM content, and Spd content compared to wild-type plants before and after drought stress. Drought treatment significantly increased *SAMS* gene expression and SAM and Spd content in WT plants. However, the OE lines showed an even greater increase, leading to a significant difference between the two groups (Figure 5c–e). Additionally, measurements of the EL (Figure 5f), RWC (Figure 5g), MDA content (Figure 5h), $O_{2}^{-}$ content (Figure 5i), H_2_O_2_ content (Figure 5j), and T-AOC (Figure 5k) indicated that the *TrMYB33* overexpression in *A. thaliana* improved plants’ resistance to drought.

### 2.6. TrMYB33 Promotes SAMS Expression and Spd Synthesis in Trifolium Repens and Improves Its Drought Tolerance

**Figure 6:**
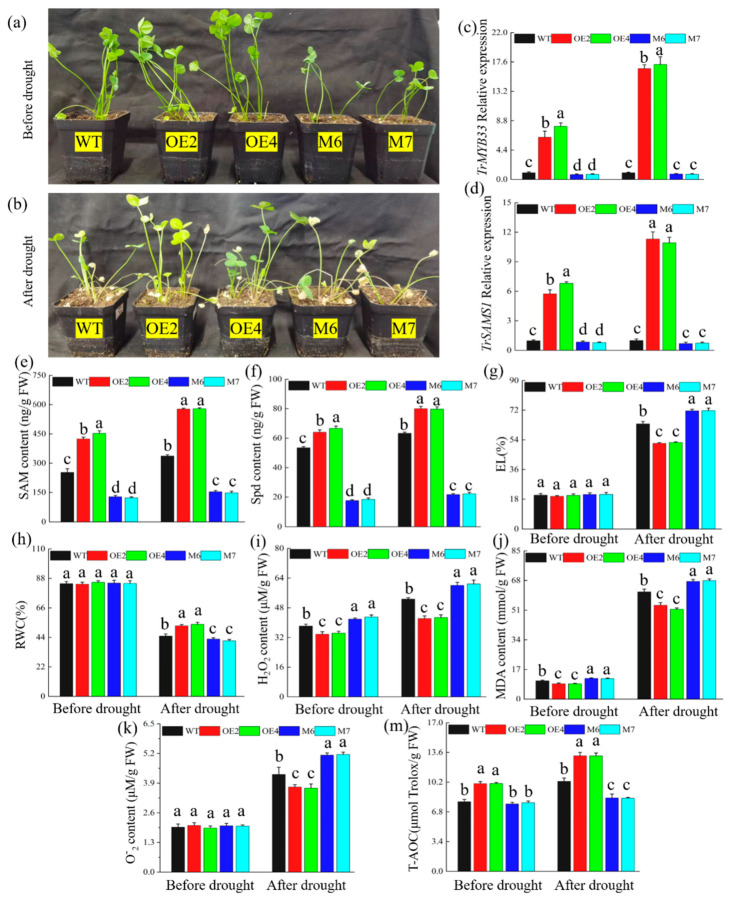
Overexpression of *TrMYB33* confers enhanced drought tolerance in transgenic *Trifolium repnes*. (**a**) Phenotype of white clover before drought stress. (**b**) Phenotype of white clover after drought stress. (**c**,**d**) Relative expression of *TrMYB33* (**c**) and *TrSAMS1* (**d**). (**e**,**f**) Content of SAM (**e**) and Spd (**f**). (**g**) EL (%). (**h**) RWC (%). (**i**) H_2_O_2_ content. (**j**) MDA content. (**k**) $O_{2}^{-}$ content. (**m**) T-AOC. Error bars indicate standard deviation (n = 3). Different letters indicate significant difference (*p* < 0.05).

In a procedure similar to that used for the generation of *Tr*SAMS1-overexpressing and -knockdown *T. repens* plants, we also generated *Tr*MYB33-overexpressing and -knockdown *T. repens* lines. For their generation, we continuously cultivated a white clover callus that emitted green fluorescence under excitation light and that eventually produced leaves and roots to obtain the OE transgenic lines. In our subsequent experiments, we utilized two of the resulting *Tr*MYB33-overexpressing white clover lines, OE2 and OE4, which showed the highest *Tr*MYB33 expression levels (Figure S10a). The VIGS construct was generated by recombining the *Tr*MYB33 fragment with a linearized pTRV2 plasmid. M6- and M7-knockdown lines, which had the lowest *TrMYB33* expression levels (Figure S10b), were selected for the subsequent experiments. White clover plants overexpressing *TrMYB33* were tall and sturdy, with most of their leaves remaining green even after eight days of drought, a characteristic indicative of enhanced drought tolerance. For comparison, the phenotype of white clover plants before drought is shown in Figure 6a. *TrMYB33*-knockdown white clover plants exhibited greater sensitivity, with most of their leaves turning yellow after eight days of drought (as depicted in Figure 6b). The relative expression of *TrMYB33* and *TrSAMS1* was significantly higher before and after drought stress in the OE2 and OE4 plants than in the WT and knockdown plants (see Figure 6c,d). However, *TrMYB33* and *TrSAMS1* relative expression were significantly lower in the M2- and M3-knockdown plants compared to the WT plants before stress but not after drought stress (see Figure 6c,d). Additionally, SAM and Spd contents in OE2 and OE4 plants were significantly higher before and after the drought compared to the WT and knockdown plants. 

Additionally, SAM and Spd content in WT plants were significantly higher compared to the knockdown plants (Figure 6e,f). Under normal growth conditions, EL was not significantly different among all plants, but the EL values (Figure 6g) of OE2 and OE4 were remarkably lower than that of WT plants. Furthermore, the RWCs (Figure 6h) of the M6 and M7 lines were higher than that of WT plants during drought, while their RWCs were not significantly different before drought. On the other hand, after drought, OEs had the highest RWC, and knockdown plants had the lowest. H_2_O_2_ (Figure 6i) and MDA (Figure 6j) were significantly decreased in OEs and notably increased in knockdown plants before and after drought. The $O_{2}^{-}$ content did not significantly vary among the plant lines under normal growth conditions. However, significant differences were observed between the WT plants and the OE and knockdown lines under drought conditions (Figure 6k). During normal growth, a significant difference in T-AOC was observed between the WT and overexpression plants, while no significant differences were identified compared with the knockdown lines. However, after drought stress, the difference was significant between the WT plants and the OE and knockdown lines (Figure 6m). Above all, these findings indicate that overexpression of *TrMYB33* played a key role in regulating plant drought resistance. 

According to the assessment of MYB-DNA interactions, R1R2R3-MYB proteins in plants exhibit distinct DNA-binding specificity [39,40,41]. According to Y1H experiments, 3R-MYBs, *Nt*MYBA1, NtMYBA2, and others bind to “AACGG”, also known as the myb-specific binding element. Each DNA target site for R2R3-MYB in plants has a specific binding sequence. In the initial research on the R2R3-MYB protein in maize, Grotewold et al. [42] discovered its ability to bind ACC(A/T)ACC(A/C/T) through binding-site-selection tests and EMSA. However, the majority of plant R2R3-MYB proteins can also bind to (T/C)AAC(G/T)G(A/C/T)(A/C/T), AGTTAGTTA, and (C/T)ACC(A/T)A (A/C)C sites. It is evident that the “TAACCA” sequence motif found in this study aligns with these sequence sites.

The analysis of *AtMYB33* in *Arabidopsis thaliana* revealed its strong similarity to GAMYB in barley [43]. No phenotype changes were observed in the *myb33* mutant, indicating the functional redundancy of *MYB33*. Additionally, the expression of *MYB33*: GUS fusion was observed only in newly formed anthers in flowers, not in stem, meristem, or root tips. Furthermore, suppressing *MYB33* expression in *A. thaliana* stem tips resulted in a stunted growth phenotype, indicating the essential role of MYB33 in plant development [44]. Additionally, MYB33 may play a role in regulating root length [45]. Here, we identified TrMYB33 as a transcription factor that regulates the expression of *TrSAMS1*. We demonstrated that *TrMYB33* has a function similar to that of *TrSAMS1* during drought, increasing the SAM and Spd content in white clover plants.

### 2.7. TrSAMS1 and TrMYB33 Are Induced by Drought and ABA in White Clover

**Figure 7:**
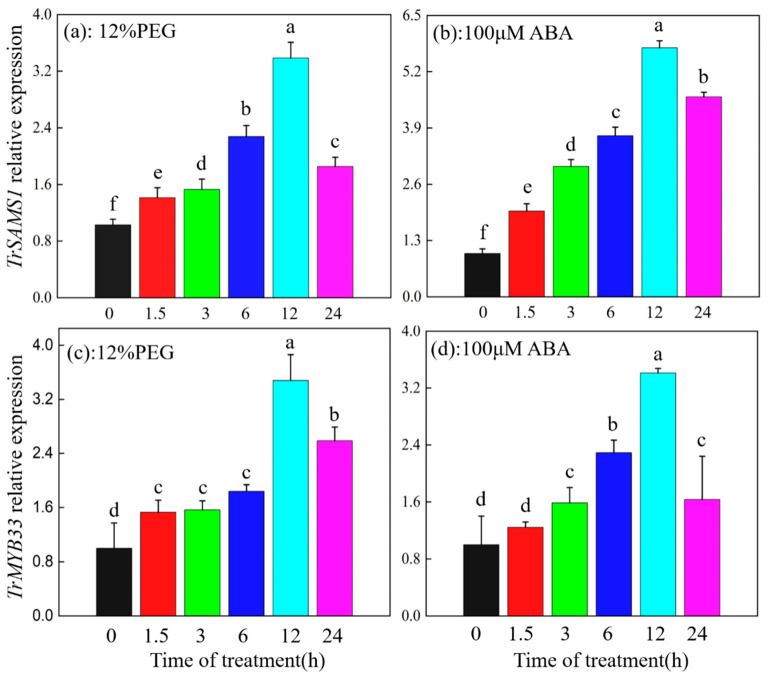
*TrSAMS1* and *TrMYB33* of *Trifolium repens* are drought-responsive genes. *TrSAMS1* and *TrMYB33* expression patterns in Ladino (*T. repens*) exposed to 12% PEG6000 treatment (**a**,**c**) and 100 μM ABA (**b**,**d**) for the designated time, as analyzed by qPCR. Error bars indicate standard deviation (n = 3). Different letters indicate significant difference (*p* < 0.05).

To examine the expression patterns of *TrSAMS1* and *TrMYB33*, we analyzed their transcript levels in the leaves of Ladino, a variety of white clover, at designated time points: 0 h, 1.5 h, 3 h, 6 h, 12 h, and 24 h. Relative expression levels of *TrSAMS1* and *TrMYB33* were determined using *TrActin101* as the internal reference. After drought-stress treatment, it was observed that both *TrSAMS1* (Figure 7a) and *TrMYB33* (Figure 7c) exhibited a slight increase in relative expression in the leaves at 1.5 h, which was followed by a progressive induction at 12 h and, subsequently, a reduction at 24 h. Such variability suggests a significant change in the expression levels of *TrSAMS1* under drought conditions. Treatment of white clover with 100 μM ABA resulted in similar changes in the relative expression of *TrSAMS1* (Figure 7b) and *TrMYB33* (Figure 7d). 

### 2.8. Drought Induces Spd Accumulation and Exogenous Spd Enhances Drought Tolerance of White Clover

**Figure 8:**
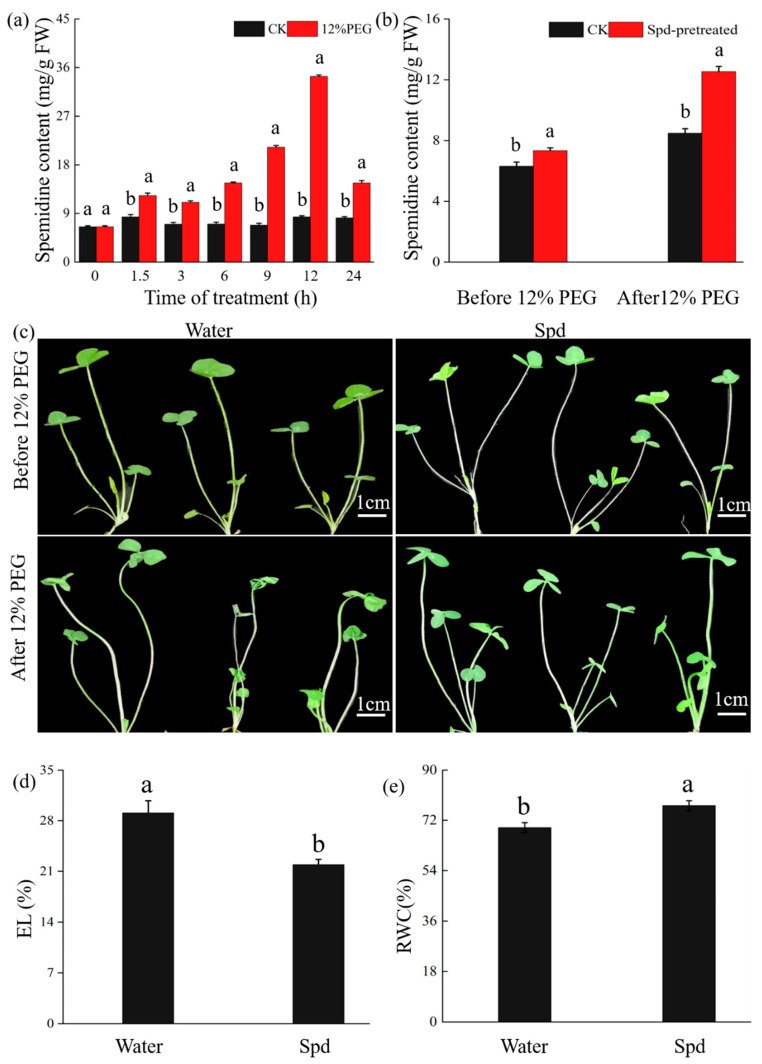
Drought induced Spd accumulation and exogenous Spd enhanced drought tolerance of white clover. (**a**) Endogenous spermidine content in white clover shoots at designated time points of drought treatment. (**b**) Spermidine level and (**c**) phenotype of water or spermidine (10 mM)-pretreated (1 d) 30-day-old seedlings before and after 12% PEG6000 treatment (1 d). Bars, 1 cm. (**d**) EL (%) and (**e**) RWC (%) of water- or spermidine-treated (2 d) plants, measured after 12% PEG6000 treatment (1 d). Different letters indicated significant differences in value at *p* < 0.05. Error bars represent SD (n = 3).

We measured the levels of endogenous Spd in *T. repens* after treatment with 12% PEG6000. Spd levels significantly increased after 1.5 h and reached their maximum at 12 h of PEG6000 treatment (Figure 8a). Furthermore, exogenous Spd supplementation significantly increased the endogenous Spd content in comparison to the control (Figure 8b). It was observed that the seedlings subjected to a 12% PEG6000 treatment, after a Spd pretreatment, exhibited reduced leaf wilting and increased leaf turgor compared to the control seedlings (Figure 8c). Lower levels of EL (Figure 8d) and RWC (Figure 8e) were observed in plants pretreated with Spd. These findings suggest that drought induces Spd accumulation in *T. repens* and that exogenously applied Spd mitigates drought-induced damage.

Drought triggers oxidative stress in plants through the substantial generation of reactive oxygen species (ROS) [46,47,48]. In plants, there are three commonly occurring small, low-molecular-weight polycationic compounds called polyamines: putrescine (Put), spermidine (Spd), and spermine (Spm). Other polyamines are less frequently present [49]. In addition to mediating growth, differentiation, and cell-death processes, polyamines play a critical role in modulating plant responses to abiotic stresses [24,50,51] by scavenging free radicals and balancing osmotic pressure directly [52]. Moreover, Spd has been determined to be more tightly associated with plant stress resistance [53,54,55,56,57].

Transgenic *A. thaliana* T2-generation plants overexpressing the Spd synthetase gene from *Cucurbita ficifolia* displayed significantly enhanced Spd synthetase activity. Consequently, the levels of Spd and Spm in transgenic plants were statistically significantly increased compared to those in the wild type. This suggests that the increased activity of Spd synthetases catalyzed the increased synthesis of Spd in T2 lines. Meanwhile, the transgenic *A. thaliana* plants demonstrated enhanced drought resistance [51].

In previous experiments investigating the influence of exogenous Spd on white clover seed germination under water stress, Spd treatment enhanced the germination rate, activity, index, root activity, and length of the seeds. Additionally, Spd reduced the degree of lipid peroxidation during seed germination while increasing the activities of superoxide dismutase, peroxidase, catalase, and ascorbate peroxidase [52]. In our research, we generated *A. thaliana* plants overexpressing the *TrSAMS1* gene. Subsequently, these plants showed considerable augmentation of SAM and Spd contents in comparison to the wild type under both normal and drought growth conditions. This finding suggests that overexpression of *TrSAMS1* leads to an increase in SAM levels in *A. thaliana*, thereby promoting Spd synthesis. Additionally, overexpression of *TrSAMS1* significantly increased the expression of the *SAMS* gene, SAM content, and Spd content in white clover. Conversely, their levels were significantly reduced in the *TrSAMS1*-knockdown white clover plants, thereby confirming the inherent presence of the SAMS→SAM→Spd regulatory pathway. Consequently, both *TrSAMS1* transgenic *A. thaliana* and white clover exhibited increased resistance to drought stress by decreasing $O_{2}^{-}$, H_2_O_2_, and MDA levels, as well as EL.

### 2.9. ABA Signaling Is Involved in Spd Accumulation in White Clover under Drought Conditions

**Figure 9:**
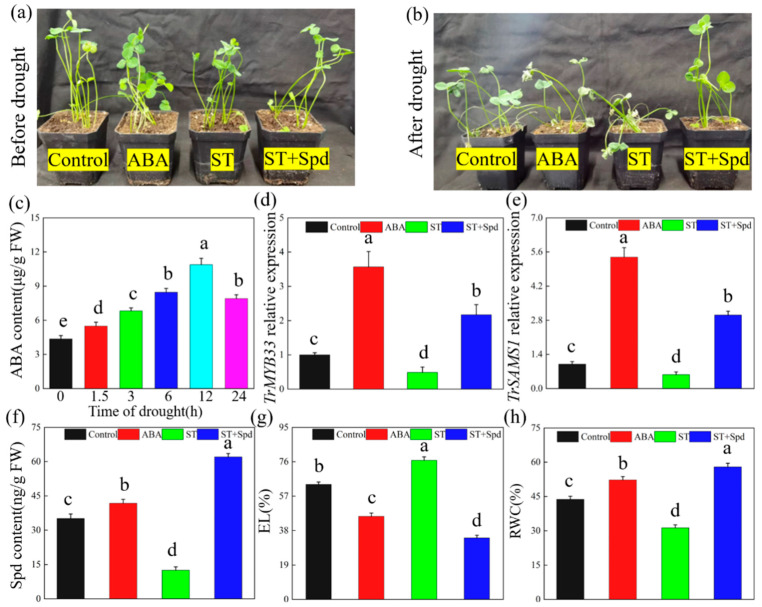
Determination of phenotypic and physiological indexes of white clover in Spd backfill experiment. Phenotype of white clover before drought (**a**) and after drought (**b**). (**c**) ABA content. (**d**) TrMYB33 relative expression and (**e**) TrSAMS1 relative expression. (**f**) Spd content. (**g**) EL (%). (**h**) RWC (%). Different letters indicated significant differences in value at *p* < 0.05.

Before the onset of drought, no discernible phenotypic differences were apparent between the treatment and the control groups (Figure 9a). Following eight days of drought stress, no significant difference in appearance was observed between the ABA treatment group and the control group. However, in the ST treatment group, the leaves of white clover plants manifested the most severe wilting. In contrast, plants in the ST + Spd treatment group showed the highest level of drought resistance (Figure 9b). We analyzed the ABA content of white clover after ABA treatment and found that the maximum ABA content was observed after 12 h of treatment (Figure 9c). We also measured the relative expression levels of *TrMYB33* and *TrSAMS1* at 12 h among all treatment groups. While the ABA treatment group exhibited significant increases in *TrMYB33* and *TrSAMS1* expression levels, the ST treatment group exhibited a significant decrease in their expression levels (Figure 9d,e). After eight days of drought treatment, the Spd content in white clover is shown in Figure 9f, while EL and RWC are shown in Figure 9g and Figure 9h, respectively. These findings indicate that the ST treatment led to more severe drought damage in white clover but that supplementation of Spd helped to alleviate this damage.

ABA plays a vital role in a wide range of plant growth and development processes, such as seed dormancy, leaf senescence, regulation of flowering, and fruit ripening, in addition to its involvement in plant responses to drought stress [58,59,60]. By regulating the stomatal aperture and controlling the expression of genes encoding for structural and regulatory proteins, including transcription-factor genes, via signal transduction pathways, ABA can effectively modulate water balance in plants under drought stress conditions. Several plant genes have been reported to be induced by exogenous ABA, as documented by Campbell et al. [61] and Yoshida et al. [62]. In *A. thaliana*, 245 genes were identified as inducible by ABA, and 299 genes were identified as inducible by drought. Of these, 155 genes were found to be inducible by both ABA and drought, indicating that ABA is involved in regulating most genes in *A. thaliana* in response to drought stress [63]. When subjecting detached leaves from different drought-tolerant alfalfa cultivars to PEG stress, Ivanova et al. [64] found that the drought-tolerant cultivars could sustain elevated levels of ABA for a prolonged time. In contrast, the drought-sensitive cultivars exhibited only a short-term increase in ABA. During water stress, the VSP protein content increased in alfalfa and white clover [65]. In addition, the VSP content also increased with different ABA-treatment concentrations [66].

## 3. Materials and Methods

### 3.1. Plant Materials and Growth Conditions

*Nicotiana benthamiana* (cv. k326) seeds were planted in plastic pots (10 cm in length and width, 15 cm in depth), filled with nutrient soil, a mixture of Pindstrup substrate and vermiculite at a ratio of 9:1 (*v*/*v*). The seeds of *T. repens* (cv. Ladino) were firstly planted in plastic boxes (30 cm in length, 25 cm in width, and 10 cm in depth) filled with quartz sand with a particle diameter of 0.3 to 0.5 cm and supplemented with Hoagland’s nutrient solution. Then, 30-day-old uniformly sized white clover plants were transplanted into plastic pots. To analyze the effect of exogenous Spd on drought responses, 30-day-old white clover plants were treated with water (as a control) or 10 mM Spd for 1 d before exposure to 12% PEG6000. To explore the expression patterns of *TrSAMS1* and *TrMYB33*, 30-day-old white clover plants were treated with 12% PEG6000 and 100 μM ABA and sampled at 1.5 h, 3 h, 6 h, 12 h, and 24 h after treatment. All samples were immediately frozen in liquid nitrogen and stored at −80 °C until further analyses. The growth chamber’s culture conditions were 12 h of light at 23 °C and 12 h of darkness at 19 °C, with approximately 220 μmol/ (m^2^·s) of photosynthetically active radiation (PAR).

### 3.2. RNA Extraction and Quantitative Real-Time RT-PCR Analysis

Total RNA was extracted using the HiPure Plant RNA Mini Kit (R4151; Guangzhou Magen Biotechnology Co., Ltd. Guangzhou, China) according to the manufacturer’s instructions. Then, first-strand complementary DNA (cDNA) was synthesized using the PrimeScript RT reagent Kit with gDNA Eraser (RR047A; Takara, Beijing, China). Quantitative real-time reverse transcription polymerase chain reaction (qRT-PCR) was conducted on a Real-Time PCR system (CFX96, Bio-rad, Hercules, CA, USA) using the NovoStart SYBR qPCR Supermix Plus (Novoprotein, Suzhou, China). *Actin2* and *Actin101* were used as internal reference genes for *A. thaliana* and *T. repens*, respectively [67]. Relative expression levels were calculated with the 2^−ΔΔCt^ formula [68]. Primers are listed in Supporting Information Table S1.

### 3.3. Isolation and Analysis of TrSAMS1

A transcriptome dataset obtained previously (not published) was analyzed to uncover differentially expressed genes (DEGs) involved in Spd biosynthesis. Three unigenes (Unigene18063, CL11265, CL6473) annotated as S-adenosylmethionine synthetases (SAMS) were upregulated 1.2-, 1.3-, and 4-fold, respectively. Then, cDNA was amplified through reverse transcription polymerase chain reaction (RT-PCR). The obtained DNA was sequenced, multiple sequence comparisons were performed, and phylogenetic trees were constructed. Phylogenetic analyses indicated that the isolated SAMS was most closely related to *AtSAMS1* of *Arabidopsis thaliana*. Thus, the gene was named *TrSAMS1* (Accession No. MH807625).

### 3.4. Construction and Screening of a cDNA Library

Total RNA was extracted from leaves of *T. repens* seedlings exposed to drought for one day and then used for constructing a prey cDNA library with the pGADT7 vector through a commercial kit (Matchmaker Gold Yeast One-Hybrid Library Screening System, Cat. no. 630491; Clontech, Mountain View, CA, USA). Three bait vectors were acquired by inserting a fragment of the *TrSAMS1* promoter into the pAbAi vector. Library screening was conducted according to the Yeast Protocols Handbook (PT3024-1; Clontech, Mountain View, CA, USA). The Y1Hgold yeast cells were cultivated for 4 d at 30 °C on solid synthetic dextrose (SD)/-Ura/-Leu medium containing 2% agar and 100 ng/mL AbA. A single colony was selected and cultivated in liquid SD/-Ura/-Leu medium. Plasmid extraction was performed from yeast cells. This step was followed by amplification using PCR and sequencing of prey fragments on pGADT7 to identify interacting TFs. The identified candidate sequence was analyzed using the TAIR database, and a phylogenetic tree was constructed. Phylogenetic analyses demonstrated that the identified TF was most closely related to *At*MYB33 of *A. thaliana*. Thus, the gene was named *TrMYB33*.

### 3.5. Subcellular Localization of the TrMYB33 Protein

The *TrMYB33* CDS without the termination codon was amplified and cloned into a frame with an enhanced green fluorescent protein (EGFP) driven by the CaMV35S promoter in the pCAMBIA3300-EGFP vector. The EHA105 carrying two distinct recombinant vectors was transiently transfected into *Arabidopsis* protoplasts [69]. In particular, for *Tr*MYB33, plasmids expressing the nuclear marker 35S: LSD1 [70] were co-transformed. Subcellular localization of the target proteins was observed using a confocal laser scanning microscope (FV10i; Olympus, Tokoyo, Japan).

### 3.6. Plasmid Construction and Plant Transformation

The full-length CDS of *TrSAMS1* or *TrMYB33* was cloned into pCAMBIA3300-EGFP under the CaMV35S promoter. The recombinant vectors were then transformed into the *Agrobacterium tumefaciens* strain EHA105, which was used to generate transgenic white clover [71] and *A. thaliana* [72] plants. Transgenic plants were selected on MS [73] medium containing 50 µg/mL kanamycin. Transgenic *A. thaliana* plants were selfed until the T3 generation was reached, while white clover plants directly derived from in vitro multiplication and the callus line were used for further analysis.

### 3.7. Virus-Induced Gene Silencing (VIGS) of TrMYB33 or TrSAMS1 in White Clover

pTRV1 (Genbank Accession No. AF166084.1) and pTRV2 (Genbank Accession No. AF406991) were used for the TRV-VIGS system, as has been reported in detail [74]. The 325 nt gene sequences of *TrPDS* (Phytoenedesaturase) and a 400 nt fragment of *TrMYB33 (*Accession No. ON435710) were inserted into the vector pTRV2. The primers for *TrPDS* were TrPDS-325-F and TrPDS-325-R, and those for *TrMYB33* were TrMYB33-400-F and TrMYB33-400-R. The final verified recombinant vectors were then transformed into the GV3101 *Agrobacterium tumefaciens* strain to conduct the VIGS experiment. To this end, an OD_600_ of 0.6 of pTRV1/GV3101 and pTRV2/GV3101 was mixed at a volume ratio of 1:1.

### 3.8. Yeast One-Hybrid (Y1H) Assay

In the present study, a yeast one-hybrid (Y1H) assay was performed according to the manufacturer’s protocol (Clontech, Mountain View, CA, USA). The 1885bp sequence upstream of *TrSAMS1* corresponding to its promoter region was cloned. According to the distribution of MYB binding sites in the promoter region of *TrSAMS1*, four partial promoter fragments designated as P1, P2, P3, and P4 and containing the MYB binding site elements were inserted into the pAbAi vector to construct three baits. In addition, the MYB binding-site sequences (TAACCACTAACCA) in P1 were removed to generate mP1. Meanwhile, the complete CDS of *TrMYB33* was fused with the activation domain (AD) of galactose-specific transcription enhancing factor 4 (GAL4) in the pGADT7 vector to generate the prey. Y1Hgold yeast cells carrying the vector were cultured for 5 d at 30 °C on SD/-Ura/-Leu medium with or without 100 ng/mL AbA.

### 3.9. Dual Luciferase Assay

The full-length CDS of *TrMYB33* was inserted into the pGreenII 62-SK vector to generate the effector, and the P1 and P1-M fragments of the *TrSAMS1* promoter were inserted into the pGreenII0800-LUC vector to generate reporters. The reporter and effector constructs were co-transformed into *A. tumefaciens* EHA105 (pSoup) and were used to infiltrate six-week-old *N. benthamiana* leaves. Transient expression was assessed by determining firefly luciferase (LUC) and Renilla luciferase (REN) luciferase activities through the Duo-Lite Luciferase Assay System (DD1205-01, Vazyme, Nanjing, China) on a microplate reader (Varioskan LUX; Thermofisher, Waltham, MA, USA). Four biological replicates were prepared for each sample.

### 3.10. Super-Shift Electrophoretic Mobility Shift Assay (EMSA)

The full-length CDS of *TrMYB33* was cloned into pGBKT7 to generate a fusion protein with the tag Myc-TrMYB33, which was subsequently expressed in *Escherichia coli* BL21 (DE3). After growing in an LB liquid medium for 12 h at 37 °C, the total proteins were extracted by using a Beyolytic Bacterial active protein-extraction reagent (P0013Q, Beyotime, Shanghai, China) according to the manufacturer’s instructions. A 22-bp single-strand DNA fragment containing the wild-type or mutated MYB binding sites was synthesized based on the P1 sequence and labeled using an EMSA probe biotin labeling kit (GS008, Beyotime, Shanghai, China). At the same time, fragments without biotin labeling were used as competitors. Super-shift EMSA was conducted using the Chemiluminescent EMSA Kit (GS009, Beyotime, Shanghai, China) complemented with washing liquid (GS009W, Beyotime, Shanghai, China), test-balancing fluid (GS009A, Beyotime, Shanghai, China), and sealing fluid (GS009B, Beyotime, Shanghai, China). Subsequently, the protein–DNA complexes were separated on 6% native-polyacrylamide gel, electroblotted onto a nylon membrane (FFN10, Beyotime, Shanghai, China), crosslinked under an ultraviolet lamp for 10 min, sealed and washed on the nylon membrane, and then visualized using chemiluminescence on ChemiDoc imaging system (Bio-Rad, Hercules, CA, USA).

### 3.11. Transcriptional Activation Assay for TrMYB33

Based on the location of the MYB binding domain in *TrMYB33*, the complete CDS of *TrMYB33* and two truncated fragments (N-terminal and C-terminal regions) were separately inserted into the pGBKT7 vector (Clontech, Mountain View, CA, USA) to generate constructs and to assess the *TrMYB33* transcriptional activation activity. An AH109 yeast strain with an α-galactosidase (MEL1) reporter gene was transformed with the constructs and incubated on SD/-Trp, SD/-Trp/-Ade, and SD/-Trp/-Ade/-His/X-α-gal (20 mg/L). The pGBKT7 vector was used as the negative control, and the pGBKT7-53 + pGADT7-T vector was used as the positive control.

### 3.12. Drought Tolerance Assays

All posts were filled with soil of identical weight and were watered thoroughly before stress, and stress conditions were identical. For drought tolerance assessment, three-week-old *A. thaliana* wild-type (WT) and transgenic plants (overexpressing *TrSAMS1* or *TrMYB33*) were treated with 12% PEG6000 for 4 d. Next, four-week-old white clovers were subjected to natural drought by water withholding (WT, *TrSAMS1*-overexpressing lines, *TrMYB33*-overexpressing lines, *TrSAMS1*-VIGS lines, *TrMYB33*-VIGS lines) for eight days. Leaves were collected at the designated time points for physiological analyses before and after drought treatment. Three replicates were assessed for each line and time point.

### 3.13. Physiological Measurements

Electrolyte leakage (EL) was determined as described in a previous report [75]. Shortly, ~0.1 g leaves were coated with tissue and placed into 50 mL of centrifugation tubes containing 30 mL deionized water, and the same volume of deionized water was used as the control. The tubes were shaken at a rate of 50 rpm on a thermostatic shaker (THZ-D; Peiying Experimental Equipment Co., Ltd., Suzhou, China) for 60 min at 25 °C before the first conductance was determined (S1 for the sample and SC1 for the control) using a conductivity meter (DDSJ-319L, INESA Scientific Instrument Co., Ltd., Shanghai, China). The tubes were then heated for 15 min in boiling water and subsequently naturally cooled down to 25 °C before the second conductance reading (S2 and SC2). Relative conductance was used to represent EL and calculated using RS(%) = (S1 − SC1)/(S2 − SC2) × 100. 

For determination of relative water content (RWC), about 0.1 g of fresh leaves were sampled, and this value was recorded as the fresh weight (FW). Then, leaves were dipped into distilled water for 24 h at 4 °C to obtain the saturated fresh weight (SW), then dried for 30 min at 105 °C and 48 h at 75 °C. After 3 d, dry weight (DW) was recorded. RWC was obtained using the following calculation: RWC(%) = (FW − DW)/(SW − DW) × 100%.

The $O_{2}^{-}$ content was determined according to the method of Elstner et al. [76]: 0.1 g of fresh leaves was crushed with 1.5 mL of 65 mM PBS buffer (pH = 7.8), and the slurry was centrifuged at 10,000 rpm for 15 min. Then, 0.5 mL of the supernatant (the control was 0.5 mL of PBS buffer) was added to 0.5 mL of pH = 7.8 PBS buffer and 0.1 mL of hydroxylamine hydrochloride (10 mM) and incubated in a 25 °C water bath for 20 min. Then, 1 mL of sulfanilamide (58 mM) and 1 mL of α-naphthylamine (7 mM) were added, and the solution was incubated again in a 25 °C water bath for 20 min. Next, 3 mL of chloroform was then added and the samples were mixed thoroughly and centrifuged at 10,000 rpm for 5 min. The upper layer of the aqueous phase (pink) was collected to determine its absorbance at 530 nm.

For the determination of H_2_O_2_ content [77], 0.1 g of fresh white clover leaves were collected, and after the addition of 1 mL of 0.1% trichloroacetic acid (TCA), they were well ground with a plant-tissue grinder and centrifuged at 12,000 rpm for 15 min. Next, 0.5 mL of pH = 7.0 PBS buffer (10 mM) and 1 mL of 1 M potassium iodide were added to 0.5 mL of the supernatant (control was 0. 1% TCA), and the samples were shaken well and placed in the dark for 10 min. Subsequently, their absorbance at 390 nm was determined.

Approximately 0.1 g of leaves was placed in a 2 mL centrifugation tube and ground into powder on a high-throughput tissue grinder (SCIENTZ-48, Ningbo Xinzhi Biotechnology Co., Ltd., Ningbo, China). Next, 1 mL of phosphate buffer solution (PBS, 50 mM, pH 7.2) was added, and the solution was mixed by vortexing. The homogenate extract was centrifuged for 10 min at 10,000 rpm at 4 °C, and the supernatant was used for malondialdehyde (MDA) measurement using an MDA kit (G0110W; Grace Biotechnology Co., Ltd., Suzhou, China). Total protein contents were measured via the staining method with Coomassie Brilliant Blue G-250 [78]. Absorbance was measured on a spectrophotometer (UV-2700, Shimadzu, Kyoto, Japan). Total antioxidant capacity (T-AOC) was measured using an assay kit (ml094998, Mlbio, Shanghai, China) according to the instructions.

### 3.14. Quantification of SAM, Spd and ABA Content

SAMS activity was indirectly represented by the production of SAM. SAM, spermidine, and ABA contents were performed by the method of enzyme-linked immunosorbent assay (ELISA). About 0.1 g of leaves were homogenized with 1 mL of pre-cooled PBS buffer (50 mM buffer, pH 7.2) and centrifuged at 10,000 rpm for 10 min at 4 °C. The supernatant was used for quantifying through specific detection kits (ml077300 for SAM, ml077200 for spermidine, and ml077235 for ABA, Mlbio, Shanghai, China), according to the instructions of the kits. In brief, 10 µL of each sample and 40 µL dilution buffer were added to the wells of the ELISA plate, incubated, and washed, then a 50 µL enzyme-labeled reagent was added to each well. Subsequently, the plate was incubated and washed again. Then, chromogenic reagents A and B were successively added to the wells, mixed, and incubated in the dark at 37 °C for 10 min. Before readings were taken at 450 nm (OD_450_) on the microplate reader (SpectraMax190, Frederick, MD, USA), the reaction was stopped by the addition of 50 µL termination buffer provided in the kit. 

### 3.15. Exogenous ABA and Spermidine Backfill Experiment

Wild-type white clover was used as the experimental material. The ABA content of white clover was determined after treatment with 100 μM ABA for 24 h. According to the previous results, white clover treated with 100 μM ABA for 12 h, white clover treated with 100 μM sodium tungstate (ABA synthesis inhibitor, ST) for 12 h, and white clover treated with 100 μM ST + 100 μM Spd for 12 h were selected. The relative expression levels of *TrMYB33* and *TrSAMS1* were measured. After 8 d of drought treatment, the Spd content, EL, and RWC content of each treatment group were measured.

### 3.16. Statistical Analysis

All the data were processed through Origin 2021 software and displayed with means ± standard deviation. Statistical differences were determined through ANOVA based on Fisher’s least significant difference (LSD) at a significance level of *p* < 0.05.

## 4. Conclusions

**Figure 10:**
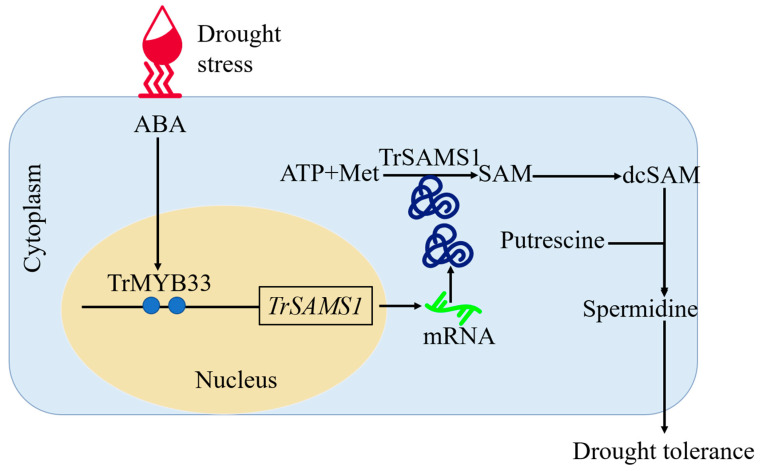
A proposed working model for illustrating the regulatory role of abscisic acid (ABA)-responsive TrMYB33-TrSAMS1 module in spermidine (Spd) accumulation under drought stress. Drought increases endogenous ABA, which subsequently starts the ABA signaling-transduction pathway by promoting the release and activation of TrMYB33. As a result, TrMYB33 consecutively positively regulates the expression of *TrSAMS1* by directly and specifically binding to the MYB binding sites and activating the promoter, bringing about upregulation of TrSAMS1, which is then joined into the metabolic pathway to promote Spd production. The “TAACCA” MYB binding site is shown in blue circle; mRNA of *TrSAMS1* is shown as a green line; while the TrSAMS1 proteins are shown using the blue curved lines.

Our study demonstrated that expression of *TrSAMS1* and *TrMYB33* were induced by drought stress and ABA. We also propose an informative and comprehensive model for understanding how Spd accumulates under drought stress, as follows: drought exposure upregulates ABA biosynthetic genes, leading to increased endogenous ABA synthesis, and TrMYB33 is induced by ABA. TrMYB33 attaches to the promoter of *TrSAMS1*, activating its expression, which promotes SAM synthesis resulting from ABA elicitation (see Figure 10). SAM then participates in and facilitates Spd biosynthesis. This model can provide a more comprehensive understanding of the molecular mechanism and transcriptional network that lead to the accumulation of Spd in response to drought. Our research also provides new insights into the role of TrMYB33-orchestrated ABA signaling in regulating Spd synthesis. TrMYB33-TrSAMS1 constitutes a transcriptional module responsible for plant ABA and drought-induced Spd synthesis, as evidenced by our findings.

## Data Availability

The data will be available on request.

## Supplementary Materials

The following supporting information can be downloaded at: https://www.mdpi.com/article/10.3390/ijms25136974/s1.
