# Supplementary material for: The White Clover TrMYB33-TrSAMS1 Module Contributes to Drought Tolerance by Modulation of Spermidine Biosynthesis via an ABA-Dependent Pathway
Source: Int J Mol Sci. 2024 Jun 26;25(13):6974. doi: 10.3390/ijms25136974 (PMC11241196; doi:10.3390/ijms25136974)
Supplement: Supplementary file 1 [file ijms-25-06974-s001.zip › ijms-3021831-supplementary.pdf]

Supplementary file

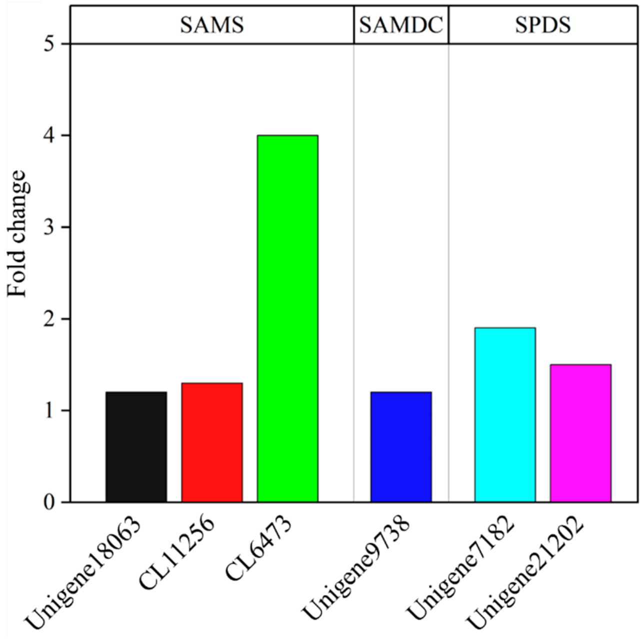

Figure S1. Expression of the six unigenes from RNA-seq dataset of white clover under drought stress, based on Fold change. SAMS: S-adenosylmethionine synthetase. SAMDC: S-adenosylmethionine decarboxylase. SPDS: spermidine synthase.

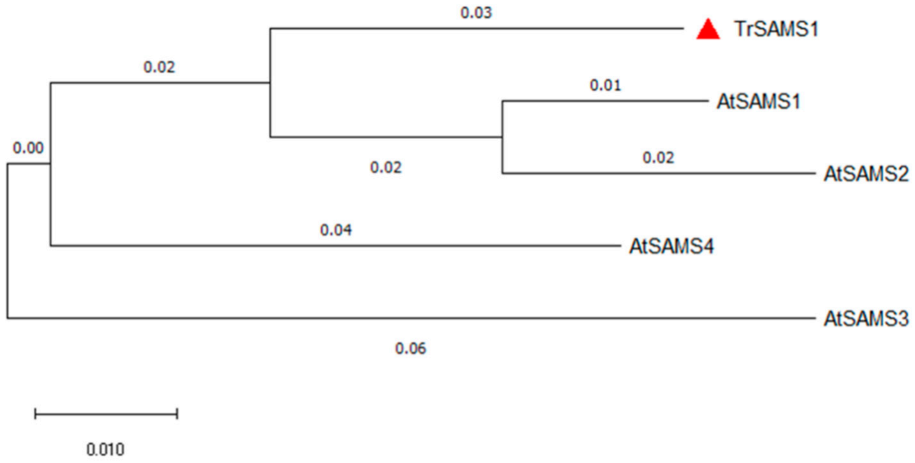

Figure S2. Phylogenetic analysis of TrSAMS1 protein and *Arabidopsis thaliana* SAMS, collected from NCBI. Number indicates branch length.
(*AtSAMS1*, NC_003070.9， AT1G02500) (*AtSAMS2*, NC_003075.7， AT4G01850)

(*AtSAMS3*, NC_003071.7， AT2G36880) (*AtSAMS4*, NC_003074.8， AT3G17390)

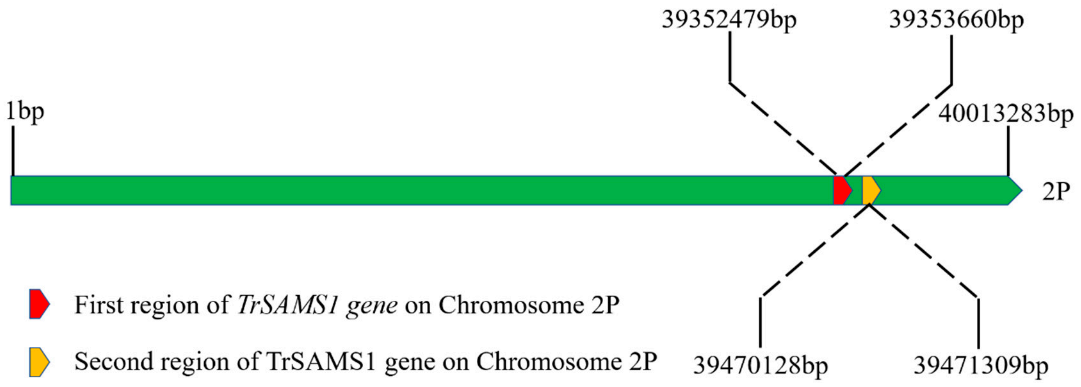

Figure S3. Schematic diagrams of matching result for *TrSAMS1* to white clover genome. The arrows are pointing in the direction of 5′ to 3′. *TrSAMS1* matched to the plus strain of 2P chromosome.

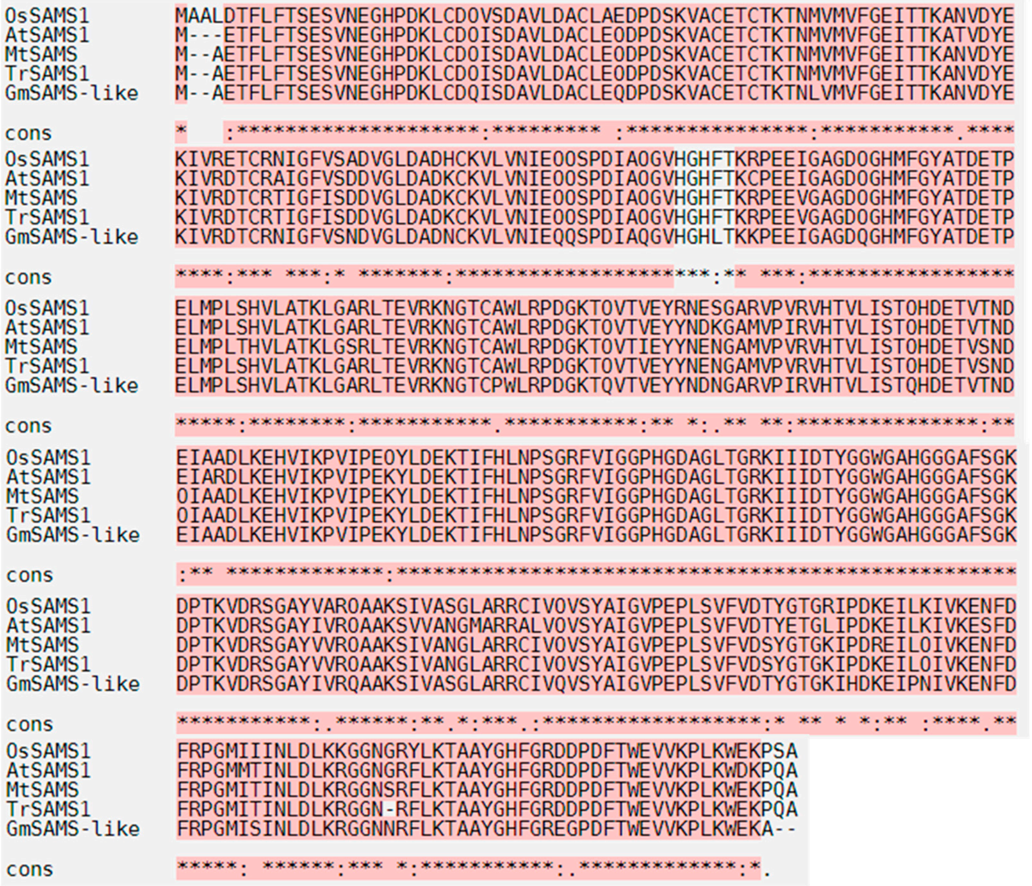

Figure S4. Protein multiple sequence alignment between TrSAMS1 and SAMS(or SAMS-like) from *Oryza sativa subsp. japonica*(OsSAMS1), *Arabidopsis thaliana*(AtSAMS1), *Medicago truncatula*(MtSAMS1), and *Glycine max*(GmSAMS-like)

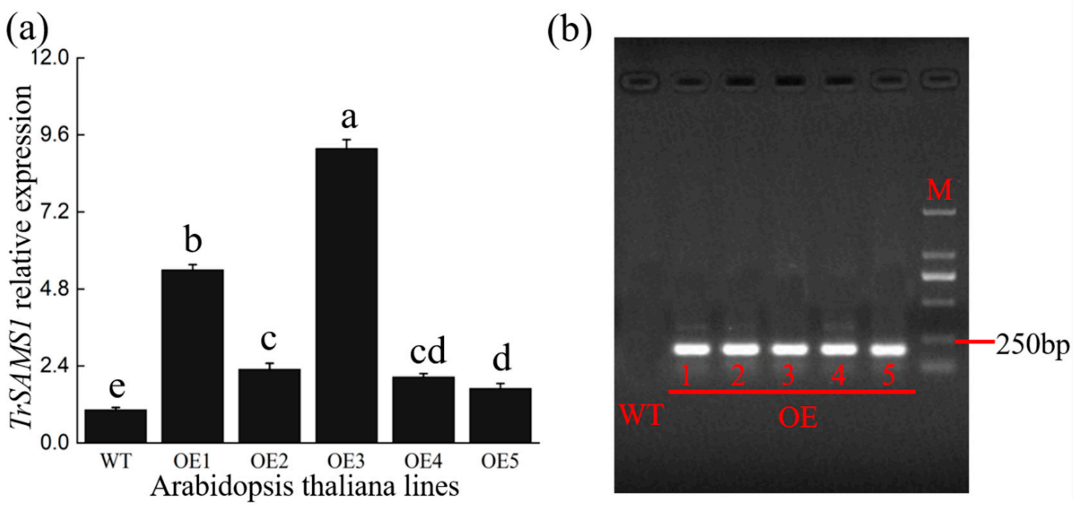

Figure S5. Overexpression of *TrSAMS1* in transgenic *A. thialiana*. (a) Analysis of *TrSAMS1* expression level in wild type (WT) and transgenic *A. thaliana*. (b) NPT gene characterization of transgenic *A.thaliana* by genomic PCR amplification.

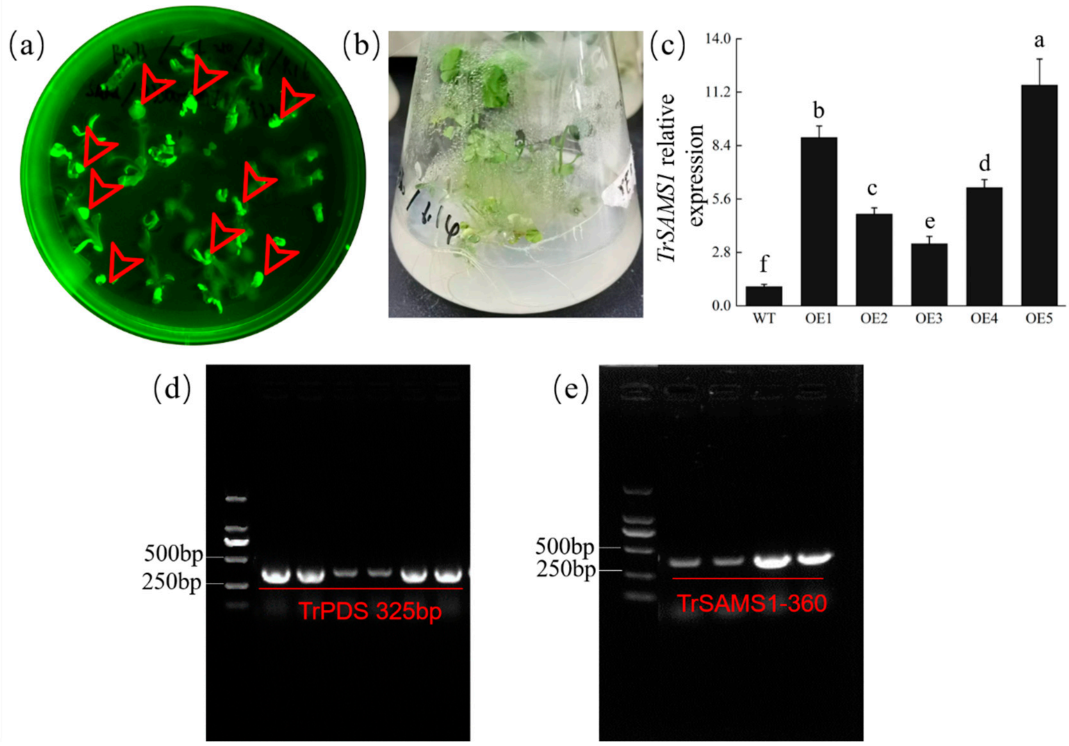

Figure S6. The acquisition of *TrSAMS1*-overexpressing white clovers and DNA fragments detection for pTRV-VIGS experiment. (a) Green fluorescence screening for callus of white clover. (b) Plant regeneration of white clover calli. (c) Analysis of *TrSAMS1* expression level in wild type (WT) and transgenic *Trifolium repens*. (d, e) Electrophoresis detection of *TrPDS* DNA fragment(c) and *TrSAMS1* DNA fragment(d) used for recombination with linearized pTRV2 carrier.

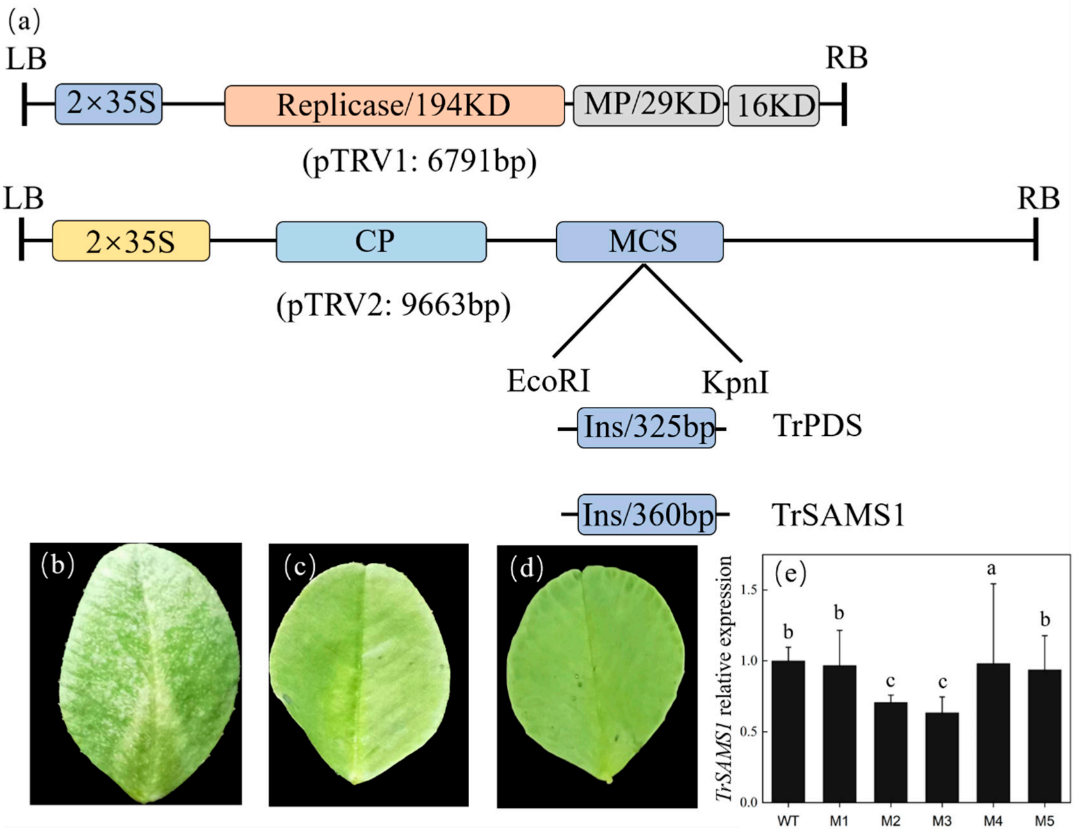

Figure S7. Schematic diagrams of constructing vectors for pTRV-VIGS system and leaves phenotypes of white clover. (a) Schematic diagrams for pTRV-VIGS system. (b, c, d). Light bleaching(b) produced by *TrPDS*-pTRV2(Positive), mock(c) and negative control(d).

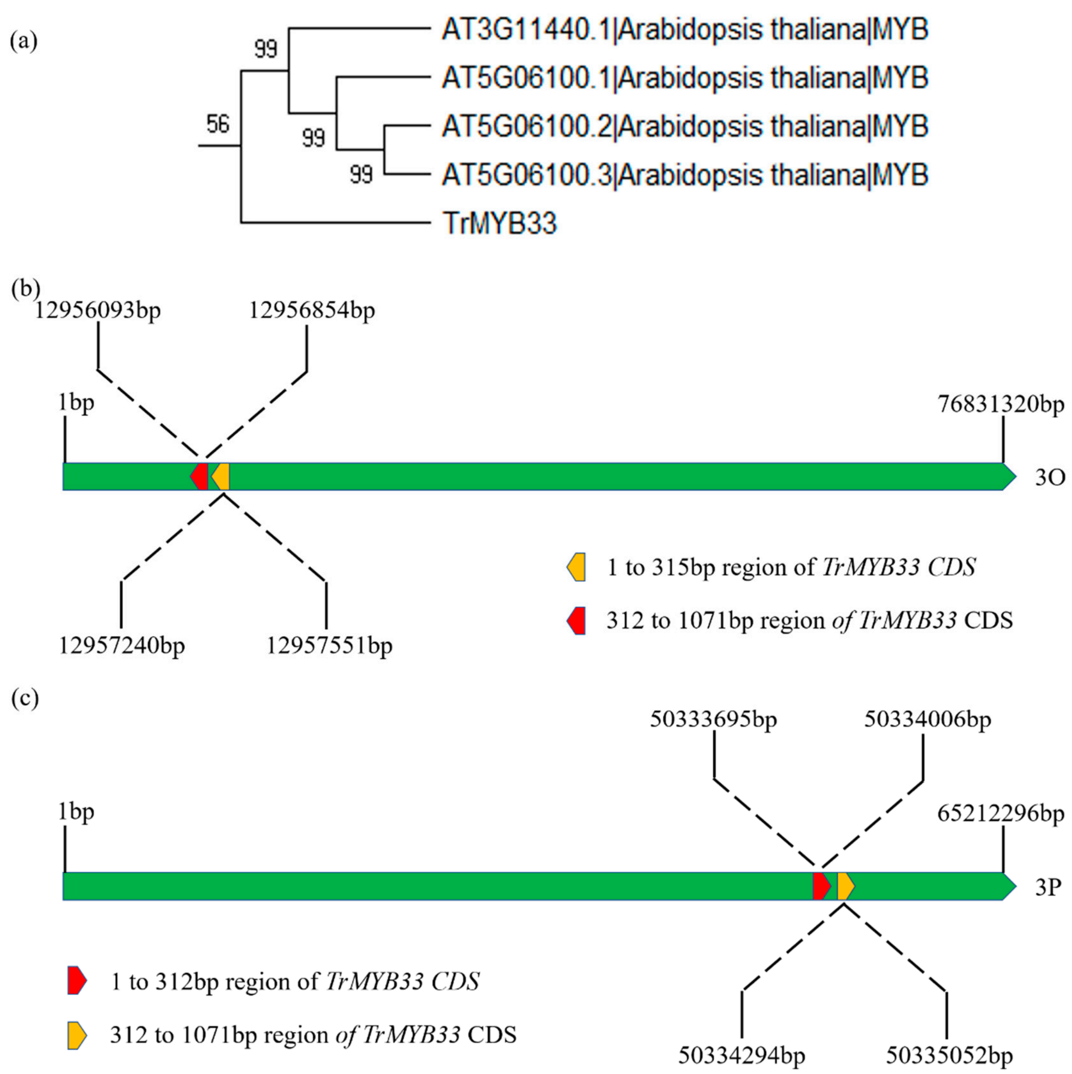

Figure S8. Phylogenetic tree of *TrMYB33* and schematic diagrams of matching result for it to white clover genome. The arrows are pointing in the direction of 5′ to 3′.(a) *TrMYB33* matched to the minus strain of 3O chromosome. (b) *TrMYB33* matched to the plus strain of 3P chromosome.

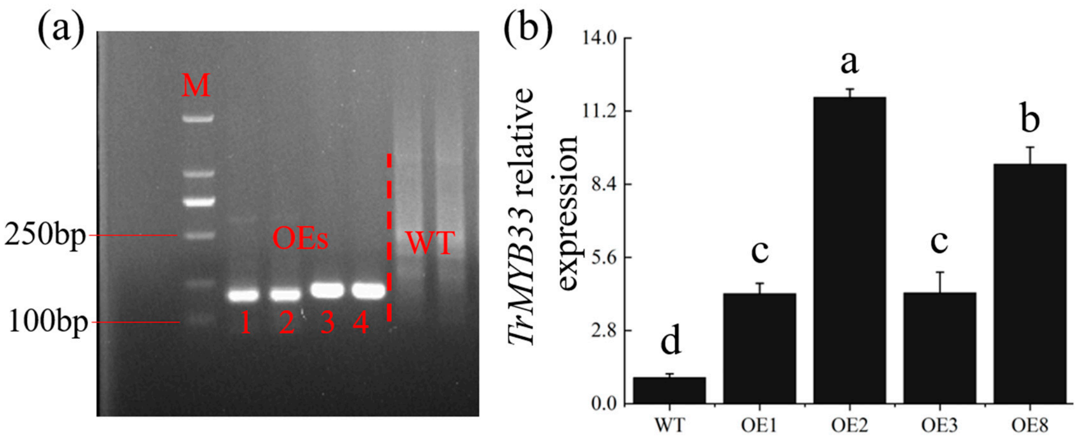

Figure S9. Overexpression of TrMYB33 in transgenic *A. thialiana*. (a) NPT gene characterization (210bp) of transgenic *A.thaliana* by genomic PCR amplification. (b) Analysis of TrMYB33 expression level in wild type (WT) and transgenic *A. thaliana*.

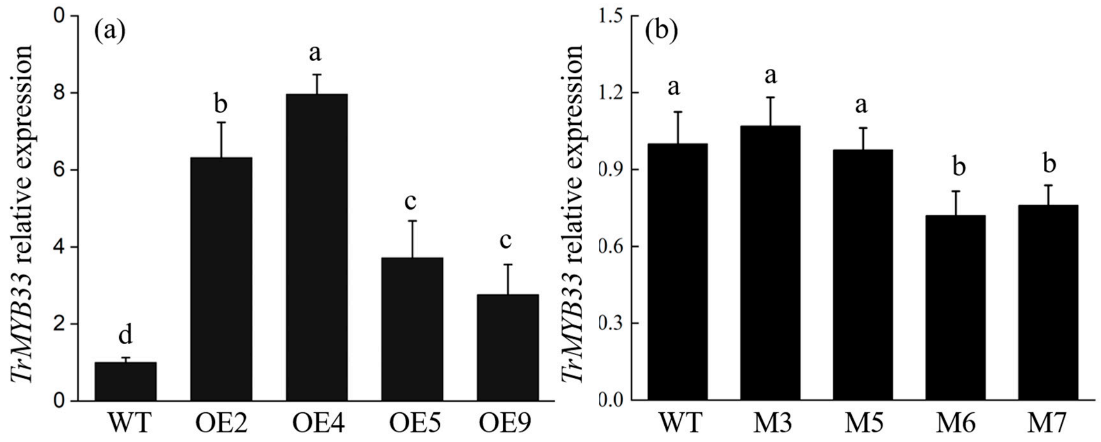

Figure S10. *TrMYB33* expression in transgenic *T. repens* and mutant *T. repens*. (a) transgenic *T. repens*. (b) mutant *T. repens.*

Table S1 List of primers used in this study.

Primers	Sequence(5'→ 3')
TrActin101-F	TGCTTGATTCCGGTGATGGTGTG
TrActin101-R	TTCTCGGCAGAGGTACTGAAGGAG
AtActin2-F	CCATCCTCCGTCTTGACCTT
AtActin2-R	CCATCCTCCGTCTTGACCTT
NPT-F	CGCAGAAGGCAATGTCATAC
NPT-R	ATTGCGAAAACTGGGAAGAA
TrMYB33-400-F	GTGAGTAAGGTTACCGAATTCATGGCTGCTTTGTT ACCTGG
TrMYB33-400-R	GAGACGCGTGAGCTCGGTACCAAGAGAGTGGTGA AGAAGATGAC
TrPDS-325-F：	GTGAGTAAGGTTACCGAATTCATGGCTCTTTCTTCACCAAAC
TrPDS-325-R：	GAGACGCGTGAGCTCGGTACCCACCAGCAATAACAACCTTC
TrSAMS1-360-F：	TGAGTAAGGTTACCGAATTCACCCTTCTGGCCGCTTTGT
TrSAMS1-360-R：	ACGCGTGAGCTCGGTACCCCAAGTTAATGGTGATCATTC
